# Supplementary material for: Genetic predisposition to high blood pressure and out-of-office hypertension: Insights from a population sample in liechtenstein
Source: PLoS One. 2026 Aug 10;21(8):e0354952. doi: 10.1371/journal.pone.0354952 (PMC13456266; doi:10.1371/journal.pone.0354952)
Supplement: Supplementary file 1 [file pone.0354952.s002.docx]

**Supplementary Table 1:** Original PROOF-BP Model (1)

|  | Prediction Model With Interaction Terms Coefficients | |
| --- | --- | --- |
|  | Systolic Blood Pressure  Prediction Model | Diastolic Blood Pressure  Prediction Model |
|  | *Beta* | *Beta* |
| Age, y | 0.07 | −0.08 |
| Sex, (men) | 3.41 | 3.33 |
| First Clinic Blood Pressure Reading | −0.50 | −0.47 |
| Change Between Clinic Blood Pressure Reading 1-3 | 0.36 | 0.31 |
| BMI | −0.21 | −0.07 |
| Hypertension Diagnosis | −5.07 | −0.03 |
| Time from Hypertension Diagnosis | 0.18 | … |
| On an Antihypertensive | 6.94 | 2.37 |
| History of cardiac disease | … | −0.40 |
| Pulse Pressure of first reading | −0.04 | −0.06 |
| Age×Clinic blood pressure | −0.01 | … |
| Age×Pulse Pressure | 0.01 | … |
| Age×Change in Clinic Blood Pressure | … | 0.01 |
| Age×BMI | … | 0.01 |
| Age× History of cardiac disease | … | 0.18 |
| Age× On an Antihypertensive | … | −0.13 |
| Sex×BMI | 0.30 | … |
| Sex×Time from Hypertension Diagnosis | −0.26 | … |
| Sex× On an Antihypertensive | −14.74 | −8.00 |
| Sex× Hypertension Diagnosis | 13.39 | 4.63 |
| Constant | −9.09 | −6.98 |

**Supplementary Bibliography**

1.	Sheppard James P., Stevens Richard, Gill Paramjit, Martin Una, Godwin Marshall, Hanley Janet, et al. Predicting Out-of-Office Blood Pressure in the Clinic (PROOF-BP). Hypertension. 2016 May 1;67(5):941–50.
