# Supplementary material for: Genetic predisposition to high blood pressure and out-of-office hypertension: Insights from a population sample in liechtenstein
Source: PLoS One. 2026 Aug 10;21(8):e0354952. doi: 10.1371/journal.pone.0354952 (PMC13456266; doi:10.1371/journal.pone.0354952)
Supplement: Supplementary file 2 [file pone.0354952.s003.docx]

**Supplementary Table 2:** Blood Pressure Phenotype Definitions

| **Phenotype** | **Definition** |
| --- | --- |
|  |  |
| Sustained Hypertension | Positive in-office hypertension and positive out-of-office hypertension |
| White Coat Hypertension | Positive in-office hypertension, normotensive out-of-office, without sustained elevation in either systolic or diastolic blood pressure |
| Masked Hypertension | Normotensive in-office, positive out-of-office hypertension, without sustained elevation in either systolic or diastolic blood pressure or white coat effects in either systolic or diastolic blood pressure |
| Normotension | No signs of sustained, white-coat, or masked-hypertension |

Out-of-Office Hypertension = defined as daytime ambulatory blood pressure of greater than or equal to 135 mmHg systolic blood pressure or 85 mmHg diastolic blood pressure; In-office Hypertension = defined as in-office blood pressure greater than or equal to 140 mmHg systolic blood pressure or 90 mmHg diastolic blood pressure for the average of the last two in-office reading
