# Supplementary material for: Genetic predisposition to high blood pressure and out-of-office hypertension: Insights from a population sample in liechtenstein
Source: PLoS One. 2026 Aug 10;21(8):e0354952. doi: 10.1371/journal.pone.0354952 (PMC13456266; doi:10.1371/journal.pone.0354952)
Supplement: Supplementary file 3 [file pone.0354952.s004.docx]

**Supplementary Table 3:** Association Between Genetic Predisposition to Elevated Blood Pressure and Blood Pressure Traits with each model adjusted for hypertension risk factors and mutually adjusted for family history and polygenic risk score

|  |  | **Systolic Blood Pressure** | | | **Diastolic Blood Pressure** | | |
| --- | --- | --- | --- | --- | --- | --- | --- |
| Exposure | Outcome | Beta | 95% CI^†^ | p-value | Beta | 95% CI^†^ | p-value |
| **PRS**^*^ | Average Office Blood Pressure Reading | 1.6 | 1.1, 2.1 | <0.001 | 1.3 | 0.9,1.7 | <0.001 |
| **PRS**^*^ | Ambulatory Daytime Blood Pressure Reading | 2.1 | 1.6, 2.5 | <0.001 | 1.5 | 1.1, 1.9 | <0.001 |
|  |  | **Systolic Blood Pressure** | | | **Diastolic Blood Pressure** | | |
| Exposure | Outcome | Beta | 95% CI^†^ | p-value | Beta | 95% CI^†^ | p-value |
| **Family History** | Average Office Blood Pressure Reading | 2.9 | 1.9, 3.9 | <0.001 | 2.1 | 1.4, 2.9 | <0.001 |
| **Family History** | Ambulatory Daytime Blood Pressure Reading | 1.9 | 0.9, 2.8 | <0.001 | 1.4 | 0.6, 2.1 | <0.001 |

^*^Polygenic Risk Score = PRS. Beta represents per 1 standard deviation increase. Systolic specific PRS effects represented for systolic blood pressure outcome. Diastolic specific PRS effects represented for diastolic blood pressure outcome.

^†^CI = Confidence Interval; All family history and polygenic risk score models presented above adjusted for cube root of age, sex, BMI, smoking status, LDL, Triglycerides, HDL, and were mutually adjusted for PRS and FHx, respectively
