# Supplementary material for: Genetic predisposition to high blood pressure and out-of-office hypertension: Insights from a population sample in liechtenstein
Source: PLoS One. 2026 Aug 10;21(8):e0354952. doi: 10.1371/journal.pone.0354952 (PMC13456266; doi:10.1371/journal.pone.0354952)
Supplement: Supplementary file 4 [file pone.0354952.s005.docx]

**Supplementary Table 4:** Variation Explained (R^2^) by Blood Pressure Genetic Score and Family History with Office/Ambulatory Blood Pressure Traits

|  | Genetic Score | | Family History | |
| --- | --- | --- | --- | --- |
| **Blood Pressure Trait** | **Systolic BP Variation Explained** | **Diastolic BP Variation Explained** | **Systolic BP Variation Explained** | **Diastolic BP Variation Explained** |
| Average Office Blood Pressure Reading | 1.5% | 2.6% | 0.75% | 1.25% |
| Ambulatory Daytime Blood Pressure Reading | 2.6% | 3.2% | 0.18% | 0.76% |
