# Supplementary material for: Genetic predisposition to high blood pressure and out-of-office hypertension: Insights from a population sample in liechtenstein
Source: PLoS One. 2026 Aug 10;21(8):e0354952. doi: 10.1371/journal.pone.0354952 (PMC13456266; doi:10.1371/journal.pone.0354952)
Supplement: Supplementary file 5 [file pone.0354952.s006.docx]

**Supplementary Table 5:** Model Performance Compared to Office Measurements

|  | **Ambulatory Systolic Blood Pressure** | | | **Ambulatory Diastolic Blood Pressure** | | |
| --- | --- | --- | --- | --- | --- | --- |
| **Model** | **Adj R2** | **AIC** | **RMSE** | **Adj R2** | **AIC** | **RMSE** |
| Average Office BP | 53.1% | 10161 | 8.14 | 42.1% | 9480 | 6.43 |
| Average Office BP + PRS | 53.6% | 10147 | 8.10 | 42.6% | 9467 | 6.40 |
| Average Office BP + Fam Hx | 53.1% | 10161 | 8.14 | 42.0% | 9481 | 6.43 |

Average Office BP = average of last two blood pressure readings; Fam Hx = Family History; PRS = Polygenic risk score with each score fit to its corresponding phenotype i.e. diastolic blood pressure gene score fit with ambulatory diastolic blood pressure phenotype and systolic blood pressure gene score fit with ambulatory systolic blood pressure phenotype; RMSE = Root Mean Squared Error; AIC: Akaike information criterion; Adj R2 = Adjusted R-squared
