# Supplementary material for: Genetic predisposition to high blood pressure and out-of-office hypertension: Insights from a population sample in liechtenstein
Source: PLoS One. 2026 Aug 10;21(8):e0354952. doi: 10.1371/journal.pone.0354952 (PMC13456266; doi:10.1371/journal.pone.0354952)
Supplement: Supplementary file 6 [file pone.0354952.s008.docx]

We thank the editorial board for this opportunity to respond to their comments and the opportunity to have this paper rigorously reviewed. Please see our responses below regarding editorial and reviewer concerns.

----------------------------------------------------------------------------------------------------------------------------------

Journal Requirements:

When submitting your revision, we need you to address these additional requirements.

**1. Please ensure that your manuscript meets PLOS ONE's style requirements, including those for file naming.** The PLOS ONE style templates can be found at

<https://journals.plos.org/plosone/s/file?id=wjVg/PLOSOne_formatting_sample_main_body.pdf> and

[https://journals.plos.org/plosone/s/file?id=ba62/PLOSOne_formatting_sample_title_authors_affiliations.pdf](https://nam12.safelinks.protection.outlook.com/?url=https%3A%2F%2Ftrack.editorialmanager.com%2FCL0%2Fhttps%3A%252F%252Fjournals.plos.org%252Fplosone%252Fs%252Ffile%253Fid%3Dba62%252FPLOSOne_formatting_sample_title_authors_affiliations.pdf%2F1%2F010f019d65b86b24-d478731f-6146-48ee-af6d-5f03fc650d13-000000%2FRQc5MYVxLjwyc7rghVAzXf9HKU5bESFuevPkKgepPd8%3D257&data=05%7C02%7Csukrit.narula%40yale.edu%7C4cd4a04ad6b14fe8982d08de944b6bfa%7Cdd8cbebb21394df8b4114e3e87abeb5c%7C0%7C0%7C639111249118755848%7CUnknown%7CTWFpbGZsb3d8eyJFbXB0eU1hcGkiOnRydWUsIlYiOiIwLjAuMDAwMCIsIlAiOiJXaW4zMiIsIkFOIjoiTWFpbCIsIldUIjoyfQ%3D%3D%7C0%7C%7C%7C&sdata=wtGOE5Zm6kgblwnsc8BzcshgcJ09CStWPjTKHjGafO8%3D&reserved=0)

*Response:* This has been done

**2. Please note that PLOS One has specific guidelines on code sharing for submissions in which author-generated code underpins the findings in the manuscript. In these cases, all author-generated code must be made available without restrictions upon publication of the work. Please review our guidelines at** [**https://journals.plos.org/plosone/s/materials-and-software-sharing#loc-sharing-code**](https://journals.plos.org/plosone/s/materials-and-software-sharing#loc-sharing-code) **and ensure that your code is shared in a way that follows best practice and facilitates reproducibility and reuse.**

*Response:* We can make the analytic code available on publication.

3. We note that the grant information you provided in the ‘Funding Information’ and ‘Financial Disclosure’ sections do not match.

When you resubmit, please ensure that you provide the correct grant numbers for the awards you received for your study in the ‘Funding Information’ section.

*Response:* We have double checked the funding information and altered the information to ensure consistency.

4. Thank you for stating the following financial disclosure: [GAPP study was supported by the Liechtenstein Government, the Swiss National Science Foundation, the Swiss Heart Foundation, the Swiss Society of Hypertension (PP00P3_133681 to David Conen), the University of Basel, the University Hospital Basel, the Hanela Foundation, Schiller AG, and Novartis.].

Please state what role the funders took in the study. If the funders had no role, please state: ""The funders had no role in study design, data collection and analysis, decision to publish, or preparation of the manuscript.""

If this statement is not correct you must amend it as needed.

Please include this amended Role of Funder statement in your cover letter; we will change the online submission form on your behalf.

*Response:* “The funders had no role in study design, data collection and analysis, decision to publish, or preparation of the manuscript” has now been added to the competing interest section.

5. Thank you for stating the following in the Competing Interests section: [I have read the journal's policy and the authors of this manuscript have the following competing interests: DC received advisory board fees from Abbott, outside of the current work. DC is a recipient of a PHRI career award. LR and MR are key shareholders of the Dr Risch Medical Laboratory. During the course of the project, PMS became a full-time employee at Deep Genomics and subsequently Takeda. However, his role was limited to before he began industry employment and the results/project are not related to the work he conducts in his industry role. ST holds a junior scholar award from the FRQS (Fonds de recherche du Québec–Santé). GP holds the Canada Research Chair in Genetic and Molecular Epidemiology and Cisco Systems Professorship in Integrated Health Biosystems. SN, MRC, KG, AL, SA have nothing to disclose.].

Please confirm that this does not alter your adherence to all PLOS ONE policies on sharing data and materials, by including the following statement: ""This does not alter our adherence to PLOS ONE policies on sharing data and materials.” (as detailed online in our guide for authors <http://journals.plos.org/plosone/s/competing-interests>). If there are restrictions on sharing of data and/or materials, please state these. Please note that we cannot proceed with consideration of your article until this information has been declared.

Please respond by return email with your amended Competing Interests Statement and we will change the online submission form on your behalf.

*Response:* The competing interests have no bearing on our data sharing policy. This statement (“This does not alter our adherence to PLOS ONE policies on sharing data and materials”) has been added to the competing interests section to reflect this.

6. In the online submission form you indicate that your data is not available for proprietary reasons and have provided a contact point for accessing this data. Please note that your current contact point is a co-author on this manuscript. According to our Data Policy, the contact point must not be an author on the manuscript and must be an institutional contact, ideally not an individual. Please revise your data statement to a non-author institutional point of contact, such as a data access or ethics committee, and send this to us via return email. Please also include contact information for the third party organization, and please include the full citation of where the data can be found.

*Response:* As stated in the data availability section, data underlying the results presented in the study are available from Private University of the Principality of Liechtenstein and can be reached at [irb@ufl.li](mailto:irb@ufl.li). We will incorporate this in a return email.

7. Please include captions for your Supporting Information files at the end of your manuscript, and update any in-text citations to match accordingly. Please see our Supporting Information guidelines for more information: <http://journals.plos.org/plosone/s/supporting-information>.

*Response:* This has been done

8. If the reviewer comments include a recommendation to cite specific previously published works, please review and evaluate these publications to determine whether they are relevant and should be cited. There is no requirement to cite these works unless the editor has indicated otherwise.

*Response:* Thank you for clarifying. Any citations included were done so based solely on their relevance.

[Note: HTML markup is below. Please do not edit.]

Reviewers' comments:

Reviewer's Responses to Questions

**Comments to the Author**

1. Is the manuscript technically sound, and do the data support the conclusions?

The manuscript must describe a technically sound piece of scientific research with data that supports the conclusions. Experiments must have been conducted rigorously, with appropriate controls, replication, and sample sizes. The conclusions must be drawn appropriately based on the data presented.

Reviewer #1: Yes

Reviewer #2: Partly

Reviewer #3: Yes

2. Has the statistical analysis been performed appropriately and rigorously?

Reviewer #1: Yes

Reviewer #2: I Don't Know

Reviewer #3: Yes

3. Have the authors made all data underlying the findings in their manuscript fully available?

The [PLOS Data policy](https://nam12.safelinks.protection.outlook.com/?url=http%3A%2F%2Ftrack.editorialmanager.com%2FCL0%2Fhttp%3A%252F%252Fwww.plosone.org%252Fstatic%252Fpolicies.action%2523sharing%2F1%2F010f019d65b86b24-d478731f-6146-48ee-af6d-5f03fc650d13-000000%2F1nZUi444N9Prj-EfPkROTYNRPCPaKfn1BcVEWi3LnAk%3D257&data=05%7C02%7Csukrit.narula%40yale.edu%7C4cd4a04ad6b14fe8982d08de944b6bfa%7Cdd8cbebb21394df8b4114e3e87abeb5c%7C0%7C0%7C639111249118801155%7CUnknown%7CTWFpbGZsb3d8eyJFbXB0eU1hcGkiOnRydWUsIlYiOiIwLjAuMDAwMCIsIlAiOiJXaW4zMiIsIkFOIjoiTWFpbCIsIldUIjoyfQ%3D%3D%7C0%7C%7C%7C&sdata=os9OjoeD9nGUMmtCCoahou5WiCNdISSSZJrW%2Bg%2BYVSA%3D&reserved=0) requires authors to make all data underlying the findings described in their manuscript fully available without restriction, with rare exception (please refer to the Data Availability Statement in the manuscript PDF file). The data should be provided as part of the manuscript or its supporting information, or deposited to a public repository. For example, in addition to summary statistics, the data points behind means, medians and variance measures should be available. If there are restrictions on publicly sharing data—e.g. participant privacy or use of data from a third party—those must be specified.

Our data availability statements states the following consistent with prior work using this cohort in this journal (see PMID 38446766): “Data that underlie the results reported in this paper were collected from study participants from the Principality of Liechtenstein, a very small country, where the risk of subject identification is increased due to the size of the population (less than 40,000 inhabitants). To respect data protection and to prevent the identification of participants, data access is restricted to researchers meeting the criteria for access to confidential data. Data are available from (contact: [lorenz.risch@ufl.li](mailto:lorenz.risch@ufl.li), [martin.risch@ksgr.ch](mailto:martin.risch@ksgr.ch), and [david.conen@phri.ca](mailto:david.conen@phri.ca)). Further, the data underlying the results presented in the study are available from Private University of the Principality of Liechtenstein, Institutional Review Board (9495 Triesen; [irb@ufl.li)](mailto:irb@ufl.li)).”

Reviewer #1: Yes

Reviewer #2: Yes

Reviewer #3: Yes

4. Is the manuscript presented in an intelligible fashion and written in standard English?

PLOS ONE does not copyedit accepted manuscripts, so the language in submitted articles must be clear, correct, and unambiguous. Any typographical or grammatical errors should be corrected at revision, so please note any specific errors here.

Reviewer #1: Yes

Reviewer #2: No

Reviewer #3: Yes

5. Review Comments to the Author

Please use the space provided to explain your answers to the questions above. You may also include additional comments for the author, including concerns about dual publication, research ethics, or publication ethics. (Please upload your review as an attachment if it exceeds 20,000 characters)

Reviewer #1: Manuscript # PONE-D-25-68169

In this study entitled “Genetic Predisposition to High Blood Pressure and Out-of-Office Hypertension: Insights from a Population Sample in Liechtenstein”
the authors evaluated the family history and polygenic risk scores (PRS) to measure the predisposition to hypertension. According to the results PRS provides incremental information in identification of individuals with ambulatory hypertension, unlike family history.

Comments:
Having parents, siblings, or grandparents (especially early-onset) with high blood pressure increases your own likelihood. Having one or more close family members with high blood pressure before age 60 doubles your risk. It is not solely genetic; shared family behaviors (diet, lifestyle) and genetic predisposition both play a role (Eur Heart J. 2017;38(29):2300–2308; Front. Pediatr 2018. 5:285; Nature Genetics 2019;51:51–62).

*Response to Reviewer 1:* We are in complete agreement with the reviewer that family history is not solely capturing a genetic phenomenon. This is also why we believe our finding showing PRS is uncorrelated with family history is so interesting, because it shows that there is an axis of readily measurable genetic variation that is not related at all to family history. Shared behavioral factors play an important role.

- Data regarding family history of hypertension should be collected in a detailed and accurate manner. In this study, the family history of hypertension was only reported. In this context, it is important to know the parents' age, because depending on the parents' age, blood pressure levels may not have met the criteria for hypertension according to current guidelines.

- A reported family history (FH) of hypertension may result in lower sensitivity of FH as an incremental predictor of ambulatory hypertension. Furthermore, parental age may be a confounding factor regarding this issue.

*Response to Reviewer 1:* We thank the reviewer for these astute points and are in agreement with the reviewer that truly capturing family history requires detailed multi-generational annotation as has been done in previous multigenerational studies including Framingham. We also recognize that hypertension definitions have evolved with time and that makes self-report less reliable. We think our study differs from previous studies in the pragmatic intent. In clinical practice, we do not have often have family member measurements readily available. As described by Greenland et al (PMID: 21144964), family history has more robust negative predictive value than it does positive predictive value for related conditions of MI and CHD. Within our own data, this pattern also holds true: positive predictive value for ambulatory hypertension is 39% and the negative predictive value is 65%. Hypertension family history by self report is likely even more variable given its asymptomatic status. Certainly as well, there is a difference as the reviewer has pointed out between hypertension diagnosed in a family at a younger rather than an older age. However, we would like to note that our paper did not conclude that family history was not predictive, just that it did not provide incremental improvement over ambulatory BP prediction models. In fact, having a family history of hypertension was independently associated with increased blood pressure even after risk factor adjustment. In other words, it is strongly correlated but may still not provide enough information to help us identify individuals with ambulatory hypertension beyond what is already in the established PROOF-BP model. To acknowledge these meaningful points by reviewer 1, we have added these points to the discussion section:

“Second, our measure of family history relied on participant self-report which is subject to recall bias. This may be less reliable than previous studies which rely on confirmed inter-generational ascertainment of hypertension status and can more clearly document age of onset across generations. Despite this, self-report of family history is often the only information available at the bedside and thus may be more relevant as a clinical operationalization of family history than those with family history observed and verified by study teams. Furthermore, hypertension definitions have evolved with time, which may further impact self-report. Despite these limitations, self-reported family history remains a pragmatic clinical standard that in our data still predicts out-of-office hypertension status even though it does not contribute beyond a standard clinical risk score (PROOF-BP).”

- In the conclusion of the study authors suggested “the possibility of PRS having clinical utility in future applications as methodology improves.” Considering that: "PRS provides modest incremental information in determining hypertension status outside the office in young, healthy individuals," do the authors believe that the cost-benefit ratio will be worthwhile for using the method in the clinic?

*Response to Reviewer 1:* We thank the reviewer for this very practical question. In short, we believe formal cost-utility studies will be required to truly make this determination and to make any declaration at this stage for the area of hypertension remains premature. We do note the work of Kiflen et al (PMID: 35904973) suggests the potential for cost-effectiveness for polygenic risk scores in guiding statin initiation. As genotyping costs fall, polygenic risk scores are ordered for other indications, and predictiveness inevitably changes with larger and more ancestry concordant studies, we believe the cost-effectiveness of obtaining these scores will improve with time. However, we are not yet at the stage where we can formally comment on the cost-benefit analysis and is beyond the scope of the current study.

Reviewer #2: The manuscript evaluates two measures of genetic predisposition to hypertension—family history and polygenic risk scores (PRS)—and examines their association with blood pressure traits using a validated prediction model. The authors further assess the incremental predictive value of these measures.
The study population appears well-characterized and representative. The findings may be relevant for the development of predictive models aimed at identifying individuals at increased risk of daytime ambulatory hypertension using readily available clinical and genetic information. However, the relatively limited clinical utility reported should be carefully considered.
The following comments and suggestions are intended to improve the scientific quality, clarity, and presentation of the manuscript.
Major Comments
1. Cohort Selection and Clinical Interpretation
The GAPP cohort began recruitment approximately 16 years ago. The authors should clarify the rationale for restricting the study population to individuals aged 31–40 years. Additionally, if participants were healthy at baseline, the implications of detecting hypertension (both office-based and ambulatory) during follow-up should be discussed in greater depth, particularly in terms of clinical relevance and potential early risk stratification.

*Response to Reviewer 2:* We thank the reviewer for their question and curiosity. In short, the GAPP study was intentionally focused on young and apparently healthy individuals. Midlife, in particular, individuals after 40 are very heavily studied as it relates to the evaluation of risk factors for atherosclerosis. There is a dearth of studies in this particular age group where risk factor progression is known to happen, but for which knowledge is lacking. In particular, by focusing on individuals such as the ones in GAPP, we are able to highlight risk factor progression and assess subclinical markers that may drive progression of risk factor (hypertension) development. GAPP not only examined genetic determinants, but also molecular determinants to see what could be subclinical drivers. Additionally because comorbid burden is low, the proportion of variance explained by factors like genetic risk scores should in theory be highest allowing for a prime evaluation of utility as a risk stratification marker. For further details, we would refer the reviewer to our previously published protocol and design paper (PMID: 23299990). We agree with the reviewer that clinical relevance of early detection of hypertension in young healthy populations should be discussed in greater detail. As such, we have added this section into this discussion:

“Furthermore, detecting hypertension in this group with low comorbidity burden carries meaningful clinical implications even among individuals who appear healthy at baseline. It allows for more refined cardiovascular risk factor assessment and stratification; opens the door to more aggressive risk factor modification with modification of diet, weight management, and abstinence from alcohol; and permits more structured surveillance and follow-up.”

2. Genotyping Methodology and Data Transparency
The manuscript lacks sufficient detail regarding the genotyping results obtained using the HumanCoreExome BeadChip. The authors should:
o Specify the number of unrelated individuals included in the genetic analyses.
o Report the total number of genetic markers analyzed and those excluded after quality control.
o Clarify how these data contributed to PRS construction.
Including this information would enhance reproducibility and methodological transparency.

*Response to Reviewer 2:* We find these requests to be very reasonable. We have included such information below and have now included the information as Supplementary figure 1.

3. Interpretation of Predictive Models
In Table 3, the inclusion of additional model performance metrics—such as the change in Akaike Information Criterion (ΔAIC)—is recommended to facilitate interpretation of the incremental value of PRS and family history. This would strengthen the evaluation of model improvement.

*Response to Reviewer 2:* We note this suggestion and have modified table 3 to reflect this change.

Moderate Comments
4. Clarification of Statistical Measures
In Table 2, it should be clearly stated whether the reported β coefficients represent changes in mmHg per standard deviation (SD) increase in the predictor variables.

Response to Reviewer 2: “Beta represents *change in blood pressure (in mmHg)* per 1 standard deviation increase” has now been added to the footnote in Table 2
5. Completeness of Tables and Footnotes
o Tables 3 and 4: The term “Proof BP RC” appears to be missing or incomplete and should be corrected.

*Response to Reviewer 2*: This has been updated. Thank you for pointing this out.
o Table 4: Abbreviations such as RL, Sen, and Spec should be explicitly defined in the footnote.

*Response to Reviewer 2:* LR is now defined in the footnote. Sen and spec have been fully written out and not abbreviated.
o Table 2: The sample size (n) should be included for both PRS and family history analyses.
*Response to Reviewer 2:* The sample size which is the same for both analyses is now listed in the table title.

6. Organization of Supplementary Material
The placement and referencing of supplementary material within the main text should be improved to ensure a logical and coherent flow of information.

*Response to Reviewer 2:* We have renamed the tables and updated their references to ensure logical cohrence and appropriate ordering.
Minor Comments
7. Tables and Formatting
o Table 1 should explicitly indicate whether values are presented as mean ± standard deviation or in another format.

*Response to Reviewer 2:* “Baseline characteristics were presented with median and interquartile range for continuous variables and numbers with proportions for categorical variable” has now been added to the footnote of table 1 to ensure clarity.

o Numerical ranges should be consistently formatted using hyphens (e.g., 10–20) rather than commas throughout the text and tables.

*Response to Reviewer 2:* This has been updated
o Abbreviations should be standardized (e.g., use “FHx” consistently instead of “Fam Hx” in Tables 3 and 4).

*Response to Reviewer 2:* This has been updated
8. Abstract and Keywords
The relevance and specificity of the keywords should be reviewed to ensure they accurately reflect the main topics and improve indexing.

*Response to Reviewer 2:* This has been updated
9. Citations and References
Additional citations should be incorporated where appropriate to support statements and contextualize findings within the existing literature. Attention should also be paid to correct punctuation placement (e.g., periods after parentheses in in-text citations).

*Response to Reviewer 2:* This has been updated
10. Language and Typographical Errors
The manuscript requires careful proofreading to correct typographical errors and ensure consistency in language usage throughout.

*Response to Reviewer 2:* We have since undertaken a thorough effort to proofread and fix typographical errors
Overall, the manuscript addresses a relevant topic in cardiovascular risk prediction. Addressing the points outlined above will substantially improve its clarity, methodological rigor, and interpretability.

Reviewer #3: The study aimed to investigate whether genetic predisposition can identify individuals with ambulatory daytime hypertension by analyzing family history and polygenic risk scores (PRS) in a White European sample from Liechtenstein. The authors conclude that PRS provides incremental value in identifying individuals with ambulatory hypertension, whereas family history does not. However, these improvements are modest and highlight the need for further refinement to enhance predictive utility in clinical practice.

General Comment

I agree with the authors that PRS offers only a modest improvement and that further research is warranted to enhance its predictive value at the point of care. Overall, the manuscript is well written, and the study appears to be carefully conducted.

General Question

Based on their experience, do the authors foresee a role for PRS testing in future clinical practice for hypertension, particularly in the context of both ambulatory and office-based hypertension?

*Response to Reviewer 3:* We see PRS as playing a potential role in identifying individuals who may need more intensive screening. There has been work till date suggesting that polygenic risk scores may help identify those at risk of earlier onset hypertension. The key to their implementation will be finding the right PRS for the right population. Interestingly, there appears to be prognostic information in the PRS that goes beyond blood pressure itself and is independent of traditional risk factors. In fact within our own analysis, blood pressure PRS is completely uncorrelated with indices such as BMI and triglycerides suggesting that there are separate axes on which this particularly PRS is exerting its predictive capabilities. We note that although it as of yet it does not display incremental value over current models for predicting ambulatory BP, it is still very strongly and robustly associated and points to the fact that there is room to go to better understand the pathogenesis of hypertension. We have now included the following passage in our discussion:

“ Moreover, PRS-based methods have shown promise in the realm of hypertension. An analysis of two Swedish cohorts found that a blood pressure PRS was associated with an increased *incidence* of hypertension with an effect size comparable to that of body-mass index.(19) *Another analysis of the UKBiobank showed that a PRS for office BP was associated with incident cardiovascular disease independent of measured office BP, suggesting that it may provide additional information on cardiovascular risk. In fact, within our own data, blood pressure PRS is completely uncorrelated with indices such as BMI and lipid measures suggesting that there are separate axes on which this particularly PRS is exerting its predictive capabilities*.”

Specific Questions for the Authors

How was family history of hypertension assessed? While this is often considered a simple binary variable (yes/no), it is not always accurately known. How reliable do the authors consider this commonly used measure?

*Response to Reviewer 3:* Family history in this study was classified on the basis of self-report. Truly capturing the effect of family history requires detailed multi-generational annotation. This has been done in studies like Framingham. As the reviewer points out, in clinical practice, we do not often have family member measurements readily available. As described by Greenland et al (PMID: 21144964), family history has more robust negative predictive value than it does positive predictive value for related conditions of MI and CHD. Within our own data, this also holds true (see the response to the next question).

Hypertension family history by self-report is likely even more variable given its asymptomatic status and unclear age of onset in some cases.We also recognize that hypertension definitions have evolved with time and that may also impact self-report. That being said, we recognize it is elicited in practice and that scientific statements endorse its use. We have now expanded on ramifications of self-report in the limitations section:

“Second, our measure of family history relied on participant self-report which is subject to recall bias. This may be less reliable than previous studies which rely on confirmed inter-generational ascertainment of hypertension status and can more clearly document age of onset across generations. Despite this, self-report of family history is often the only information available at the bedside and thus may be more relevant as a clinical operationalization of family history than those with family history observed and verified by study teams. Furthermore, hypertension definitions have evolved with time and that fact may also impact self-report.”

Do the authors believe it would be useful to collect more detailed information, such as the age at onset of hypertension in relatives or whether (and which) relatives were receiving antihypertensive treatment? Family history is an important risk factor, particularly when focused on first-degree relatives. Does the predictive value differ depending on whether the affected relatives are parents, siblings, or offspring? Would it be informative, for example, to investigate hypertension during pregnancy in female relatives? More broadly, does stratifying family history by sex (e.g., mother vs. father, sisters vs. brothers) add meaningful information?

*Response to Reviewer 3:* We appreciate this question. We find that sex of the family member who had hypertension is no more predictive. For those with a first degree male family member, PPV and NPV was 41% and 65%, respectively. For those with a first degree female family member, PPV and NPV was 38% and 64% respectively. Having multiple family members with hypertension did not meaningfully alter informativeness with PPV and NPV of 43% and 64%, respectively. We have now included this information in the results section of the manuscript. We believe that having detailed information including age of onset of hypertension in family members and whether relatives were receiving antihypertensive treatment could potentially add some information although we note that in the absence of primary data collection from relatives, this type of information could be subject to recall bias. We have made it a point to mention this in our limitations as referenced in the above response.

The study population is relatively young; therefore, a hypertension prevalence of 12% is noteworthy. Could stress—or any available measures of stress—have influenced the observed blood pressure values?

*Response to Reviewer 3:* We thank the reviewer for this observation. Although this number seems high, we feel this is in line with prevalence estimates in this age group from other cohorts. For example, in this analysis of representative United States data (NHANES; doi: 10.15620/cdc/164016), 23% of adults between age 18-39 were noted to have hypertension. It is then unsurprising given we have excluded those with comorbidities (such as OSA and severe obesity; see below) that our prevalence estimates may be lower. Unfortunately, while we have measured circulating biomarkers, we do not have any known markers of stress or cortisol levels.

Was any correlation observed between blood pressure and heart rate? Including this information might provide additional insight.

*Response to Reviewer 3:* We do think this information would be valuable, however, our data did not include simultaneous heart rate measurements that would allow for such correlation.

Among participants identified as hypertensive, how many were already aware of their condition?

*Response to Reviewer 3:* For our analysis, we excluded those with pre-existing hypertension (as the goal of the analysis was to identify those with out of office hypertension). Prior to this exclusion, 2.5% of participants self-identified as having hypertension.

Were comorbidities (beyond common risk factors) taken into account in the data collection and analysis?

*Response to Reviewer 3:* Comorbidity data was indeed collected across all participants using standardized forms and questionnaires. However, participants with significant comorbidities including severe obesity, renal disease, significant cardiovascular disease, and documented OSA were excluded by design. This decision was intentional: by excluding participants with conditions that independently alter the outcome of interest, increased cohort homogeneity could more cleanly capture antecedent risk.

The inclusion and exclusion criteria are referenced (ref. 11), but a brief summary in the manuscript would improve readability.

*Response to Reviewer 3:* We appreciate the efforts to improve clarity. In the manuscript, we have added inclusion and exclusion criteria in the main body of the text here: “Participants were eligible for the sample if they were residents of the Principality of Liechtenstein between the ages of 25 and 41 at baseline. They were excluded if they had a history of major illness, including cardiovascular disease, treated diabetes mellitus, renal disease, atrial fibrillation, heart failure, severe obesity (body mass index>35), or sleep apnea.”

The HumanCore Exome BeadChip by Illumina is mentioned: is this a validated tool? It would be helpful to include an appropriate reference.

*Response to Reviewer 3:* The HumanCore Exome BeadChip by Illumina is indeed a validated tool with good genome wide coverage. Our genetic quality control pipeline missingness, HWE, sex discrepancy, ancestry outlier, and relatedness filters retained 262,789 SNPs across 1,696 individuals driven almost entirely by removal of rare variants with MAF < 0.01. Despite the moniker of Exome chip, it has been used in several large common variant studies including: Sjaarda et al (PMID: 32059761) and Øvretveit et al (PMID: 39291479). It is also referenced by Choi et al (PMID: 32709988) in the text of this Nature Protocol on performing polygenic risk scores.

The following was added to the paper in the Methods section:

“Genotyping was done using the HumanCoreExome BeadChip designed by Illumina Inc (San Diego, CA), a validated genotyping chip has been used in several large common variant studies” with citations included for Sjaarda and Øvretveit papers mentioned above.
