# Supplementary material for: Genetic predisposition to high blood pressure and out-of-office hypertension: Insights from a population sample in liechtenstein
Source: PLoS One. 2026 Aug 10;21(8):e0354952. doi: 10.1371/journal.pone.0354952 (PMC13456266; doi:10.1371/journal.pone.0354952)
Supplement: Peer review history

# Peer review: Decision Letter 0

**DOI:** 10.1371/journal.pone.0354952.r001

**Authors:** Marcelo Nakazone

6 Apr 2026

PONE-D-25-68169

Genetic Predisposition to High Blood Pressure and Out-of-Office Hypertension: Insights from a Population Sample in Liechtenstein

PLOS One

Dear Dr. Narula,

Thank you for submitting your manuscript to PLOS ONE. After careful consideration, we feel that it has merit but does not fully meet PLOS ONE’s publication criteria as it currently stands. Therefore, we invite you to submit a revised version of the manuscript that addresses the points raised during the review process.

ACADEMIC EDITOR:

While the manuscript exhibits intriguing potential, it requires substantial revisions and further refinement.

Although the inherent interest in the subject matter is acknowledged, the reviewers have raised crucial concerns that must be properly addressed.

Please submit your revised manuscript by May 21 2026 11:59PM. If you will need more time than this to complete your revisions, please reply to this message or contact the journal office at plosone@plos.org. When you're ready to submit your revision, log on to https://www.editorialmanager.com/pone/ and select the 'Submissions Needing Revision' folder to locate your manuscript file.

Please include the following items when submitting your revised manuscript:

- A letter that responds to each point raised by the academic editor and reviewer(s). You should upload this letter as a separate file labeled 'Response to Reviewers'.
- A marked-up copy of your manuscript that highlights changes made to the original version. You should upload this as a separate file labeled 'Revised Manuscript with Track Changes'.
- An unmarked version of your revised paper without tracked changes. You should upload this as a separate file labeled 'Manuscript'.

If you would like to make changes to your financial disclosure, please include your updated statement in your cover letter. Guidelines for resubmitting your figure files are available below the reviewer comments at the end of this letter.

If applicable, we recommend that you deposit your laboratory protocols in protocols.io to enhance the reproducibility of your results. Protocols.io assigns your protocol its own identifier (DOI) so that it can be cited independently in the future. For instructions see: https://journals.plos.org/plosone/s/submission-guidelines#loc-laboratory-protocols. Additionally, PLOS ONE offers an option for publishing peer-reviewed Lab Protocol articles, which describe protocols hosted on protocols.io. Read more information on sharing protocols at https://plos.org/protocols?utm_medium=editorial-email&utm_source=authorletters&utm_campaign=protocols.

As the corresponding author, your ORCID iD is verified in the submission system and will appear in the published article. PLOS supports the use of ORCID, and we encourage all coauthors to register for an ORCID iD and use it as well. Please encourage your coauthors to verify their ORCID iD within the submission system before final acceptance, as unverified ORCID iDs will not appear in the published article. *Only*  the individual author can complete the verification step; PLOS staff *cannot*  verify ORCID iDs on behalf of authors.

We look forward to receiving your revised manuscript.

Kind regards,

Marcelo Arruda Nakazone, M.D., Ph.D.

Academic Editor

PLOS One

Journal Requirements:

When submitting your revision, we need you to address these additional requirements.

1. Please ensure that your manuscript meets PLOS ONE's style requirements, including those for file naming. The PLOS ONE style templates can be found at

https://journals.plos.org/plosone/s/file?id=wjVg/PLOSOne_formatting_sample_main_body.pdf and

https://journals.plos.org/plosone/s/file?id=ba62/PLOSOne_formatting_sample_title_authors_affiliations.pdf

2. Please note that PLOS One has specific guidelines on code sharing for submissions in which author-generated code underpins the findings in the manuscript. In these cases, all author-generated code must be made available without restrictions upon publication of the work. Please review our guidelines at https://journals.plos.org/plosone/s/materials-and-software-sharing#loc-sharing-code and ensure that your code is shared in a way that follows best practice and facilitates reproducibility and reuse.

3. We note that the grant information you provided in the ‘Funding Information’ and ‘Financial Disclosure’ sections do not match.

When you resubmit, please ensure that you provide the correct grant numbers for the awards you received for your study in the ‘Funding Information’ section.

4. Thank you for stating the following financial disclosure: [GAPP study was supported by the Liechtenstein Government, the Swiss National Science Foundation, the Swiss Heart Foundation, the Swiss Society of Hypertension (PP00P3_133681 to David Conen), the University of Basel, the University Hospital Basel, the Hanela Foundation, Schiller AG, and Novartis.].

Please state what role the funders took in the study.  If the funders had no role, please state: ""The funders had no role in study design, data collection and analysis, decision to publish, or preparation of the manuscript.""

If this statement is not correct you must amend it as needed.

Please include this amended Role of Funder statement in your cover letter; we will change the online submission form on your behalf.

5. Thank you for stating the following in the Competing Interests section: [I have read the journal's policy and the authors of this manuscript have the following competing interests: DC received consulting fees from Roche Diagnostics and Trimedics, and speaker fees from Servier and BMS/Pfizer, all outside of the current work. LR and MR are key shareholders of the Dr Risch Medical Laboratory. During the course of the project, PMS became a full-time employee at Deep Genomics and subsequently Takeda. However, his role was limited to before he began industry employment and the results/project are not related to the work he conducts in his industry role. ST holds a junior scholar award from the FRQS (Fonds de recherche du Québec–Santé). GP holds the Canada Research Chair in Genetic and Molecular Epidemiology and Cisco Systems Professorship in Integrated Health Biosystems. SN, MRC, KG, AL, SA have nothing to disclose.].

Please confirm that this does not alter your adherence to all PLOS ONE policies on sharing data and materials, by including the following statement: ""This does not alter our adherence to  PLOS ONE policies on sharing data and materials.” (as detailed online in our guide for authors http://journals.plos.org/plosone/s/competing-interests). If there are restrictions on sharing of data and/or materials, please state these. Please note that we cannot proceed with consideration of your article until this information has been declared.

Please respond by return email with your amended Competing Interests Statement and we will change the online submission form on your behalf.

6. In the online submission form you indicate that your data is not available for proprietary reasons and have provided a contact point for accessing this data. Please note that your current contact point is a co-author on this manuscript. According to our Data Policy, the contact point must not be an author on the manuscript and must be an institutional contact, ideally not an individual. Please revise your data statement to a non-author institutional point of contact, such as a data access or ethics committee, and send this to us via return email. Please also include contact information for the third party organization, and please include the full citation of where the data can be found.

7. Please include captions for your Supporting Information files at the end of your manuscript, and update any in-text citations to match accordingly. Please see our Supporting Information guidelines for more information: http://journals.plos.org/plosone/s/supporting-information.

8. If the reviewer comments include a recommendation to cite specific previously published works, please review and evaluate these publications to determine whether they are relevant and should be cited. There is no requirement to cite these works unless the editor has indicated otherwise.

[Note: HTML markup is below. Please do not edit.]

Reviewers' comments:

Reviewer's Responses to Questions

**Comments to the Author**

1. Is the manuscript technically sound, and do the data support the conclusions?

The manuscript must describe a technically sound piece of scientific research with data that supports the conclusions. Experiments must have been conducted rigorously, with appropriate controls, replication, and sample sizes. The conclusions must be drawn appropriately based on the data presented.

Reviewer #1: Yes

Reviewer #2: Partly

Reviewer #3: Yes

**********

2. Has the statistical analysis been performed appropriately and rigorously?

Reviewer #1: Yes

Reviewer #2: I Don't Know

Reviewer #3: Yes

**********

3. Have the authors made all data underlying the findings in their manuscript fully available?

The PLOS Data policy requires authors to make all data underlying the findings described in their manuscript fully available without restriction, with rare exception (please refer to the Data Availability Statement in the manuscript PDF file). The data should be provided as part of the manuscript or its supporting information, or deposited to a public repository. For example, in addition to summary statistics, the data points behind means, medians and variance measures should be available. If there are restrictions on publicly sharing data—e.g. participant privacy or use of data from a third party—those must be specified.

Reviewer #1: Yes

Reviewer #2: Yes

Reviewer #3: Yes

**********

4. Is the manuscript presented in an intelligible fashion and written in standard English?

PLOS ONE does not copyedit accepted manuscripts, so the language in submitted articles must be clear, correct, and unambiguous. Any typographical or grammatical errors should be corrected at revision, so please note any specific errors here.

Reviewer #1: Yes

Reviewer #2: No

Reviewer #3: Yes

**********

5. Review Comments to the Author

Please use the space provided to explain your answers to the questions above. You may also include additional comments for the author, including concerns about dual publication, research ethics, or publication ethics. (Please upload your review as an attachment if it exceeds 20,000 characters)

Reviewer #1: Manuscript # PONE-D-25-68169

In this study entitled “Genetic Predisposition to High Blood Pressure and Out-of-Office Hypertension: Insights from a Population Sample in Liechtenstein”

the authors evaluated the family history and polygenic risk scores (PRS) to measure the predisposition to hypertension. According to the results PRS provides incremental information in identification of individuals with ambulatory hypertension, unlike family history.

Comments:

Having parents, siblings, or grandparents (especially early-onset) with high blood pressure increases your own likelihood. Having one or more close family members with high blood pressure before age 60 doubles your risk. It is not solely genetic; shared family behaviors (diet, lifestyle) and genetic predisposition both play a role (Eur Heart J. 2017;38(29):2300–2308; Front. Pediatr 2018. 5:285; Nature Genetics 2019;51:51–62).

- Data regarding family history of hypertension should be collected in a detailed and accurate manner. In this study, the family history of hypertension was only reported. In this context, it is important to know the parents' age, because depending on the parents' age, blood pressure levels may not have met the criteria for hypertension according to current guidelines.

- A reported family history (FH) of hypertension may result in lower sensitivity of FH as an incremental predictor of ambulatory hypertension. Furthermore, parental age may be a confounding factor regarding this issue.

- In the conclusion of the study authors suggested “the possibility of PRS having clinical utility in future applications as methodology improves.” Considering that: "PRS provides modest incremental information in determining hypertension status outside the office in young, healthy individuals," do the authors believe that the cost-benefit ratio will be worthwhile for using the method in the clinic?

Reviewer #2: The manuscript evaluates two measures of genetic predisposition to hypertension—family history and polygenic risk scores (PRS)—and examines their association with blood pressure traits using a validated prediction model. The authors further assess the incremental predictive value of these measures.

The study population appears well-characterized and representative. The findings may be relevant for the development of predictive models aimed at identifying individuals at increased risk of daytime ambulatory hypertension using readily available clinical and genetic information. However, the relatively limited clinical utility reported should be carefully considered.

The following comments and suggestions are intended to improve the scientific quality, clarity, and presentation of the manuscript.

Major Comments

1. Cohort Selection and Clinical Interpretation

The GAPP cohort began recruitment approximately 16 years ago. The authors should clarify the rationale for restricting the study population to individuals aged 31–40 years. Additionally, if participants were healthy at baseline, the implications of detecting hypertension (both office-based and ambulatory) during follow-up should be discussed in greater depth, particularly in terms of clinical relevance and potential early risk stratification.

2. Genotyping Methodology and Data Transparency

The manuscript lacks sufficient detail regarding the genotyping results obtained using the HumanCoreExome BeadChip. The authors should:

o Specify the number of unrelated individuals included in the genetic analyses.

o Report the total number of genetic markers analyzed and those excluded after quality control.

o Clarify how these data contributed to PRS construction.

Including this information would enhance reproducibility and methodological transparency.

3. Interpretation of Predictive Models

In Table 3, the inclusion of additional model performance metrics—such as the change in Akaike Information Criterion (ΔAIC)—is recommended to facilitate interpretation of the incremental value of PRS and family history. This would strengthen the evaluation of model improvement.

Moderate Comments

4. Clarification of Statistical Measures

In Table 2, it should be clearly stated whether the reported β coefficients represent changes in mmHg per standard deviation (SD) increase in the predictor variables.

5. Completeness of Tables and Footnotes

o Tables 3 and 4: The term “Proof BP RC” appears to be missing or incomplete and should be corrected.

o Table 4: Abbreviations such as RL, Sen, and Spec should be explicitly defined in the footnote.

o Table 2: The sample size (n) should be included for both PRS and family history analyses.

6. Organization of Supplementary Material

The placement and referencing of supplementary material within the main text should be improved to ensure a logical and coherent flow of information.

Minor Comments

7. Tables and Formatting

o Table 1 should explicitly indicate whether values are presented as mean ± standard deviation or in another format.

o Numerical ranges should be consistently formatted using hyphens (e.g., 10–20) rather than commas throughout the text and tables.

o Abbreviations should be standardized (e.g., use “FHx” consistently instead of “Fam Hx” in Tables 3 and 4).

8. Abstract and Keywords

The relevance and specificity of the keywords should be reviewed to ensure they accurately reflect the main topics and improve indexing.

9. Citations and References

Additional citations should be incorporated where appropriate to support statements and contextualize findings within the existing literature. Attention should also be paid to correct punctuation placement (e.g., periods after parentheses in in-text citations).

10. Language and Typographical Errors

The manuscript requires careful proofreading to correct typographical errors and ensure consistency in language usage throughout.

Overall, the manuscript addresses a relevant topic in cardiovascular risk prediction. Addressing the points outlined above will substantially improve its clarity, methodological rigor, and interpretability.

Reviewer #3: The study aimed to investigate whether genetic predisposition can identify individuals with ambulatory daytime hypertension by analyzing family history and polygenic risk scores (PRS) in a White European sample from Liechtenstein. The authors conclude that PRS provides incremental value in identifying individuals with ambulatory hypertension, whereas family history does not. However, these improvements are modest and highlight the need for further refinement to enhance predictive utility in clinical practice.

General Comment

I agree with the authors that PRS offers only a modest improvement and that further research is warranted to enhance its predictive value at the point of care. Overall, the manuscript is well written, and the study appears to be carefully conducted.

General Question

Based on their experience, do the authors foresee a role for PRS testing in future clinical practice for hypertension, particularly in the context of both ambulatory and office-based hypertension?

Specific Questions for the Authors

How was family history of hypertension assessed? While this is often considered a simple binary variable (yes/no), it is not always accurately known. How reliable do the authors consider this commonly used measure?

Do the authors believe it would be useful to collect more detailed information, such as the age at onset of hypertension in relatives or whether (and which) relatives were receiving antihypertensive treatment?

Family history is an important risk factor, particularly when focused on first-degree relatives. Does the predictive value differ depending on whether the affected relatives are parents, siblings, or offspring?

Would it be informative, for example, to investigate hypertension during pregnancy in female relatives? More broadly, does stratifying family history by sex (e.g., mother vs. father, sisters vs. brothers) add meaningful information?

The study population is relatively young; therefore, a hypertension prevalence of 12% is noteworthy. Could stress—or any available measures of stress—have influenced the observed blood pressure values?

Was any correlation observed between blood pressure and heart rate? Including this information might provide additional insight.

Among participants identified as hypertensive, how many were already aware of their condition?

Were comorbidities (beyond common risk factors) taken into account in the data collection and analysis?

The inclusion and exclusion criteria are referenced (ref. 11), but a brief summary in the manuscript would improve readability.

The HumanCore Exome BeadChip by Illumina is mentioned: is this a validated tool? It would be helpful to include an appropriate reference.

**********

6. PLOS authors have the option to publish the peer review history of their article (what does this mean?). If published, this will include your full peer review and any attached files.

If you choose “no”, your identity will remain anonymous but your review may still be made public.

**Do you want your identity to be public for this peer review?**  For information about this choice, including consent withdrawal, please see our Privacy Policy.

Reviewer #1: **Yes:** Heno Ferreira Lopes

Reviewer #2: **Yes:** Maria Cristina Moran Moguel

Reviewer #3: No

**********

[NOTE: If reviewer comments were submitted as an attachment file, they will be attached to this email and accessible via the submission site. Please log into your account, locate the manuscript record, and check for the action link "View Attachments". If this link does not appear, there are no attachment files.]

To ensure your figures meet our technical requirements, please review our figure guidelines: https://journals.plos.org/plosone/s/figures

You may also use PLOS’s free figure tool, NAAS, to help you prepare publication quality figures: https://journals.plos.org/plosone/s/figures#loc-tools-for-figure-preparation.

NAAS will assess whether your figures meet our technical requirements by comparing each figure against our figure specifications.

# Author comment: Author response to Decision Letter 1

**DOI:** 10.1371/journal.pone.0354952.r002

26 Jun 2026

We thank the editorial board for this opportunity to respond to their comments and the opportunity to have this paper rigorously reviewed. Please see our responses below regarding editorial and reviewer concerns.

----------------------------------------------------------------------------------------------------------------------------------

Journal Requirements:

When submitting your revision, we need you to address these additional requirements.

1. Please ensure that your manuscript meets PLOS ONE's style requirements, including those for file naming. The PLOS ONE style templates can be found at

https://journals.plos.org/plosone/s/file?id=wjVg/PLOSOne_formatting_sample_main_body.pdf and

https://journals.plos.org/plosone/s/file?id=ba62/PLOSOne_formatting_sample_title_authors_affiliations.pdf

Response: This has been done

2. Please note that PLOS One has specific guidelines on code sharing for submissions in which author-generated code underpins the findings in the manuscript. In these cases, all author-generated code must be made available without restrictions upon publication of the work. Please review our guidelines at https://journals.plos.org/plosone/s/materials-and-software-sharing#loc-sharing-code and ensure that your code is shared in a way that follows best practice and facilitates reproducibility and reuse.

Response: We can make the analytic code available on publication.

3. We note that the grant information you provided in the ‘Funding Information’ and ‘Financial Disclosure’ sections do not match.

When you resubmit, please ensure that you provide the correct grant numbers for the awards you received for your study in the ‘Funding Information’ section.

Response: We have double checked the funding information and altered the information to ensure consistency.

4. Thank you for stating the following financial disclosure: [GAPP study was supported by the Liechtenstein Government, the Swiss National Science Foundation, the Swiss Heart Foundation, the Swiss Society of Hypertension (PP00P3_133681 to David Conen), the University of Basel, the University Hospital Basel, the Hanela Foundation, Schiller AG, and Novartis.].

Please state what role the funders took in the study. If the funders had no role, please state: ""The funders had no role in study design, data collection and analysis, decision to publish, or preparation of the manuscript.""

If this statement is not correct you must amend it as needed.

Please include this amended Role of Funder statement in your cover letter; we will change the online submission form on your behalf.

Response: “The funders had no role in study design, data collection and analysis, decision to publish, or preparation of the manuscript” has now been added to the competing interest section.

5. Thank you for stating the following in the Competing Interests section: [I have read the journal's policy and the authors of this manuscript have the following competing interests: DC received advisory board fees from Abbott, outside of the current work. DC is a recipient of a PHRI career award. LR and MR are key shareholders of the Dr Risch Medical Laboratory. During the course of the project, PMS became a full-time employee at Deep Genomics and subsequently Takeda. However, his role was limited to before he began industry employment and the results/project are not related to the work he conducts in his industry role. ST holds a junior scholar award from the FRQS (Fonds de recherche du Québec–Santé). GP holds the Canada Research Chair in Genetic and Molecular Epidemiology and Cisco Systems Professorship in Integrated Health Biosystems. SN, MRC, KG, AL, SA have nothing to disclose.].

Please confirm that this does not alter your adherence to all PLOS ONE policies on sharing data and materials, by including the following statement: ""This does not alter our adherence to PLOS ONE policies on sharing data and materials.” (as detailed online in our guide for authors http://journals.plos.org/plosone/s/competing-interests). If there are restrictions on sharing of data and/or materials, please state these. Please note that we cannot proceed with consideration of your article until this information has been declared.

Please respond by return email with your amended Competing Interests Statement and we will change the online submission form on your behalf.

Response: The competing interests have no bearing on our data sharing policy. This statement (“This does not alter our adherence to PLOS ONE policies on sharing data and materials”) has been added to the competing interests section to reflect this.

6. In the online submission form you indicate that your data is not available for proprietary reasons and have provided a contact point for accessing this data. Please note that your current contact point is a co-author on this manuscript. According to our Data Policy, the contact point must not be an author on the manuscript and must be an institutional contact, ideally not an individual. Please revise your data statement to a non-author institutional point of contact, such as a data access or ethics committee, and send this to us via return email. Please also include contact information for the third party organization, and please include the full citation of where the data can be found.

Response: As stated in the data availability section, data underlying the results presented in the study are available from Private University of the Principality of Liechtenstein and can be reached at irb@ufl.li. We will incorporate this in a return email.

7. Please include captions for your Supporting Information files at the end of your manuscript, and update any in-text citations to match accordingly. Please see our Supporting Information guidelines for more information: http://journals.plos.org/plosone/s/supporting-information.

Response: This has been done

8. If the reviewer comments include a recommendation to cite specific previously published works, please review and evaluate these publications to determine whether they are relevant and should be cited. There is no requirement to cite these works unless the editor has indicated otherwise.

Response: Thank you for clarifying. Any citations included were done so based solely on their relevance.

[Note: HTML markup is below. Please do not edit.]

Reviewers' comments:

Reviewer's Responses to Questions

Comments to the Author

1. Is the manuscript technically sound, and do the data support the conclusions?

The manuscript must describe a technically sound piece of scientific research with data that supports the conclusions. Experiments must have been conducted rigorously, with appropriate controls, replication, and sample sizes. The conclusions must be drawn appropriately based on the data presented.

Reviewer #1: Yes

Reviewer #2: Partly

Reviewer #3: Yes

2. Has the statistical analysis been performed appropriately and rigorously?

Reviewer #1: Yes

Reviewer #2: I Don't Know

Reviewer #3: Yes

3. Have the authors made all data underlying the findings in their manuscript fully available?

The PLOS Data policy requires authors to make all data underlying the findings described in their manuscript fully available without restriction, with rare exception (please refer to the Data Availability Statement in the manuscript PDF file). The data should be provided as part of the manuscript or its supporting information, or deposited to a public repository. For example, in addition to summary statistics, the data points behind means, medians and variance measures should be available. If there are restrictions on publicly sharing data—e.g. participant privacy or use of data from a third party—those must be specified.

Our data availability statements states the following consistent with prior work using this cohort in this journal (see PMID 38446766): “Data that underlie the results reported in this paper were collected from study participants from the Principality of Liechtenstein, a very small country, where the risk of subject identification is increased due to the size of the population (less than 40,000 inhabitants). To respect data protection and to prevent the identification of participants, data access is restricted to researchers meeting the criteria for access to confidential data. Data are available from (contact: lorenz.risch@ufl.li, martin.risch@ksgr.ch, and david.conen@phri.ca). Further, the data underlying the results presented in the study are available from Private University of the Principality of Liechtenstein, Institutional Review Board (9495 Triesen; irb@ufl.li).”

Reviewer #1: Yes

Reviewer #2: Yes

Reviewer #3: Yes

4. Is the manuscript presented in an intelligible fashion and written in standard English?

PLOS ONE does not copyedit accepted manuscripts, so the language in submitted articles must be clear, correct, and unambiguous. Any typographical or grammatical errors should be corrected at revision, so please note any specific errors here.

Reviewer #1: Yes

Reviewer #2: No

Reviewer #3: Yes

5. Review Comments to the Author

Please use the space provided to explain your answers to the questions above. You may also include additional comments for the author, including concerns about dual publication, research ethics, or publication ethics. (Please upload your review as an attachment if it exceeds 20,000 characters)

Reviewer #1: Manuscript # PONE-D-25-68169

In this study entitled “Genetic Predisposition to High Blood Pressure and Out-of-Office Hypertension: Insights from a Population Sample in Liechtenstein”

the authors evaluated the family history and polygenic risk scores (PRS) to measure the predisposition to hypertension. According to the results PRS provides incremental information in identification of individuals with ambulatory hypertension, unlike family history.

Comments:

Having parents, siblings, or grandparents (especially early-onset) with high blood pressure increases your own likelihood. Having one or more close family members with high blood pressure before age 60 doubles your risk. It is not solely genetic; shared family behaviors (diet, lifestyle) and genetic predisposition both play a role (Eur Heart J. 2017;38(29):2300–2308; Front. Pediatr 2018. 5:285; Nature Genetics 2019;51:51–62).

Response to Reviewer 1: We are in complete agreement with the reviewer that family history is not solely capturing a genetic phenomenon. This is also why we believe our finding showing PRS is uncorrelated with family history is so interesting, because it shows that there is an axis of readily measurable genetic variation that is not related at all to family history. Shared behavioral factors play an important role.

- Data regarding family history of hypertension should be collected in a detailed and accurate manner. In this study, the family history of hypertension was only reported. In this context, it is important to know the parents' age, because depending on the parents' age, blood pressure levels may not have met the criteria for hypertension according to current guidelines.

- A reported family history (FH) of hypertension may result in lower sensitivity of FH as an incremental predictor of ambulatory hypertension. Furthermore, parental age may be a confounding factor regarding this issue.

Response to Reviewer 1: We thank the reviewer for these astute points and are in agreement with the reviewer that truly capturing family history requires detailed multi-generational annotation as has been done in previous multigenerational studies including Framingham. We also recognize that hypertension definitions have evolved with time and that makes self-report less reliable. We think our study differs from previous studies in the pragmatic intent. In clinical practice, we do not have often have family member measurements readily available. As described by Greenland et al (PMID: 21144964), family history has more robust negative predictive value than it does positive predictive value for related conditions of MI and CHD. Within our own data, this pattern also holds true: positive predictive value for ambulatory hypertension is 39% and the negative predictive value is 65%. Hypertension family history by self report is likely even more variable given its asymptomatic status. Certainly as well, there is a difference as the reviewer has pointed out between hypertension diagnosed in a family at a younger rather than an older age. However, we would like to note that our paper did not conclude that family history was not predictive, just that it did not provide incremental improvement over ambulatory BP prediction models. In fact, having a family history of hypertension was independently associated with increased blood pressure even after risk factor adjustment. In other words, it is strongly correlated but may still not provide enough information to help us identify individuals with ambulatory hypertension beyond what is already in the established PROOF-BP model. To acknowledge these meaningful points by reviewer 1, we have added these points to the discussion section:

“Second, our measure of family history relied on participant self-report which is subject to recall bias. This may be less reliable than previous studies which rely on confirmed inter-generational ascertainment of hypertension status and can more clearly document age of onset across generations. Despite this, self-report of family history is often the only information available at the bedside and thus may be more relevant as a clinical operationalization of family history than those with family history observed and verified by study teams. Furthermore, hypertension definitions have evolved with time, which may further impact self-report. Despite these limitations, self-reported family history remains a pragmatic clinical standard that in our data still predicts out-of-office hypertension status even though it does not contribute beyond a standard clinical risk score (PROOF-BP).”

- In the conclusion of the study authors suggested “the possibility of PRS having clinical utility in future applications as methodology improves.” Considering that: "PRS provides modest incremental information in determining hypertension status outside the office in young, healthy individuals," do the authors believe that the cost-benefit ratio will be worthwhile for using the method in the clinic?

Response to Reviewer 1: We thank the reviewer for this very practical question. In short, we believe formal cost-utility studies will be required to truly make this determination and to make any declaration at this stage for the area of hypertension remains premature. We do note the work of Kiflen et al (PMID: 35904973) suggests the potential for cost-effectiveness for polygenic risk scores in guiding statin initiation. As genotyping costs fall, polygenic risk scores are ordered for other indications, and predictiveness inevitably changes with larger and more ancestry concordant studies, we believe the cost-effectiveness of obtaining these scores will improve with time. However, we are not yet at the stage where we can formally comment on the cost-benefit analysis and is beyond the scope of the current study.

Reviewer #2: The manuscript evaluates two measures of genetic predisposition to hypertension—family history and polygenic risk scores (PRS)—and examines their association with blood pressure traits using a validated prediction model. The authors further assess the incremental predictive value of these measures.

The study population appears well-characterized and representative. The findings may be relevant for the development of predictive models aimed at identifying individuals at increased risk of daytime ambulatory hypertension using readily available clinical and genetic information. However, the relatively limited clinical utility reported should be carefully considered.

The following comments and suggestions are intended to improve the scientific quality, clarity, and presentation of the manuscript.

Major Comments

1. Cohort Selection and Clinical Interpretation

The GAPP cohort began recruitment approximately 16 years ago. The authors should clarify the rationale for restricting the study population to individuals aged 31–40 years. Ad

Attachment Submitted filename: PLOS One Revision Response_dc.docx

# Peer review: Decision Letter 1

**DOI:** 10.1371/journal.pone.0354952.r003

**Authors:** Marcelo Nakazone

15 Jul 2026

Genetic Predisposition to High Blood Pressure and Out-of-Office Hypertension: Insights from a Population Sample in Liechtenstein

PONE-D-25-68169R1

Dear Dr. Narula,

We’re pleased to inform you that your manuscript has been judged scientifically suitable for publication and will be formally accepted for publication once it meets all outstanding technical requirements.

Within one week, you’ll receive an e-mail detailing the required amendments. When these have been addressed, you’ll receive a formal acceptance letter and your manuscript will be scheduled for publication.

An invoice will be generated when your article is formally accepted. Please note, if your institution has a publishing partnership with PLOS and your article meets the relevant criteria, all or part of your publication costs will be covered. Please make sure your user information is up-to-date by logging into Editorial Manager at Editorial Manager® and clicking the ‘Update My Information' link at the top of the page. For questions related to billing, please contact billing support.

If your institution or institutions have a press office, please notify them about your upcoming paper to help maximize its impact. If they’ll be preparing press materials, please inform our press team as soon as possible -- no later than 48 hours after receiving the formal acceptance. Your manuscript will remain under strict press embargo until 2 pm Eastern Time on the date of publication. For more information, please contact onepress@plos.org.

Kind regards,

Marcelo Arruda Nakazone, M.D., Ph.D.

Academic Editor

PLOS One

Additional Editor Comments (optional):

Reviewers' comments:

Reviewer's Responses to Questions

**Comments to the Author**

1. If the authors have adequately addressed your comments raised in a previous round of review and you feel that this manuscript is now acceptable for publication, you may indicate that here to bypass the “Comments to the Author” section, enter your conflict of interest statement in the “Confidential to Editor” section, and submit your "Accept" recommendation.

Reviewer #1: All comments have been addressed

Reviewer #2: All comments have been addressed

**********

2. Is the manuscript technically sound, and do the data support the conclusions?

The manuscript must describe a technically sound piece of scientific research with data that supports the conclusions. Experiments must have been conducted rigorously, with appropriate controls, replication, and sample sizes. The conclusions must be drawn appropriately based on the data presented.

Reviewer #1: Yes

Reviewer #2: Yes

**********

3. Has the statistical analysis been performed appropriately and rigorously?

Reviewer #1: Yes

Reviewer #2: Yes

**********

4. Have the authors made all data underlying the findings in their manuscript fully available?

The PLOS Data policy requires authors to make all data underlying the findings described in their manuscript fully available without restriction, with rare exception (please refer to the Data Availability Statement in the manuscript PDF file). The data should be provided as part of the manuscript or its supporting information, or deposited to a public repository. For example, in addition to summary statistics, the data points behind means, medians and variance measures should be available. If there are restrictions on publicly sharing data—e.g. participant privacy or use of data from a third party—those must be specified.

Reviewer #1: Yes

Reviewer #2: Yes

**********

5. Is the manuscript presented in an intelligible fashion and written in standard English?

PLOS ONE does not copyedit accepted manuscripts, so the language in submitted articles must be clear, correct, and unambiguous. Any typographical or grammatical errors should be corrected at revision, so please note any specific errors here.

Reviewer #1: Yes

Reviewer #2: Yes

**********

6. Review Comments to the Author

Please use the space provided to explain your answers to the questions above. You may also include additional comments for the author, including concerns about dual publication, research ethics, or publication ethics. (Please upload your review as an attachment if it exceeds 20,000 characters)

Reviewer #1: Manudcript#PONE-D-25-68169R1

Comments:

- The authors did not address every question precisely. Since a database is involved, I imagine that some data may not be available.

- The results obtained add little to clinical application at this time.

- In light of the responses to all reviewers, there has been significant improvement in the writing and presentation of the results. The study opens up prospects for future research aimed at better identifying the potential genetic polymorphisms involved in the pathogenesis of hypertension.

Reviewer #2: The authors have responded promptly and satisfactorily to all my comments and observations. The modifications made and the considerations added, primarily in the discussion section, are relevant and have clarified the study.

**********

7. PLOS authors have the option to publish the peer review history of their article (what does this mean?). If published, this will include your full peer review and any attached files.

If you choose “no”, your identity will remain anonymous but your review may still be made public.

**Do you want your identity to be public for this peer review?**  For information about this choice, including consent withdrawal, please see our Privacy Policy.

Reviewer #1: No

Reviewer #2: No

**********

# Editor report: Acceptance letter

**DOI:** 10.1371/journal.pone.0354952.r004

**Authors:** Marcelo Nakazone

PONE-D-25-68169R1

PLOS One

Dear Dr. Narula,

I'm pleased to inform you that your manuscript has been deemed suitable for publication in PLOS One. Congratulations! Your manuscript is now being handed over to our production team.

At this stage, our production department will prepare your paper for publication. This includes ensuring the following:

* All references, tables, and figures are properly cited

* All relevant supporting information is included in the manuscript submission,

* There are no issues that prevent the paper from being properly typeset

You will receive further instructions from the production team, including instructions on how to review your proof when it is ready. Please keep in mind that we are working through a large volume of accepted articles, so please give us a few days to review your paper and let you know the next and final steps.

Lastly, if your institution or institutions have a press office, please let them know about your upcoming paper now to help maximize its impact. If they'll be preparing press materials, please inform our press team within the next 48 hours. Your manuscript will remain under strict press embargo until 2 pm Eastern Time on the date of publication. For more information, please contact onepress@plos.org.

You will receive an invoice from PLOS for your publication fee after your manuscript has reached the completed accept phase. If you receive an email requesting payment before acceptance or for any other service, this may be a phishing scheme. Learn how to identify phishing emails and protect your accounts at https://explore.plos.org/phishing.

If we can help with anything else, please email us at customercare@plos.org.

Thank you for submitting your work to PLOS ONE and supporting open access.

Kind regards,

PLOS ONE Editorial Office Staff

on behalf of

Professor Marcelo Arruda Nakazone

Academic Editor

PLOS One
